# Evaluation of Bisphenol A in Pregnant Women from 10 Caribbean Countries

**DOI:** 10.3390/toxics10100556

**Published:** 2022-09-22

**Authors:** Martin S. Forde, Suzanne Côté, Elhadji A. Laouan Sidi, Éric Gaudreau, Pierre Ayotte

**Affiliations:** 1 Department of Public Health & Preventive Medicine, St. George’s University, Grenada FZ818, West Indies; 2 Axe Santé des Populations et Pratiques Optimales en Santé, Centre de Recherche du CHU de Québec, Québec, QC G1V 5B3, Canada; 3 Centre de Toxicologie du Québec (CTQ), Institut National de Santé Publique du Québec (INSPQ), Québec, QC G1V 5B3, Canada

**Keywords:** Bisphenol A (BPA), biomonitoring, urine, prenatal exposure, Caribbean

## Abstract

Bisphenol A (BPA), a phenolic chemical incorporated into many plastic products, has been found to act as an endocrine disruptor that potentially is linked to adverse neurodevelopmental outcomes. Prenatal BPA concentration levels were assessed in 10 English-speaking Caribbean countries by randomly selecting 15 maternal urine samples from approximately 50 pregnant women samples collected in each island and then comparing the findings with comparable data from Canada and the U.S. BPA was detected in all samples ranging from a low geometric mean of 1.46 μg/L (St. Lucia) to a high of 4.88 μg/L (St. Kitts & Nevis). All of the Caribbean islands sampled had geometric mean concentration levels that were higher than those recorded in two Canadian biomonitoring surveys (1.26 μg/L and 0.80 μg/L) and the U.S. NHANES survey (1.39 μg/L). This first biomonitoring survey of BPA concentration levels in maternal urine samples taken from Caribbean countries clearly points to the need for Caribbean governments and public health officials to first engage in legislative and regulatory efforts to ban or minimize the importation and use of BPA products used the Caribbean and, second, to continue to conduct biomonitoring surveys so as to ensure that these laws and regulations are indeed leading to a decrease of BPA concentrations in Caribbean populations.

## 1. Introduction

The chemical 2,2-bis(4-hydroxyphenyl) propane, more commonly known as Bisphenol A (BPA), is a high-volume production phenolic chemical used to produce polycarbonate plastics that is incorporated into numerous commercial products ranging from shatterproof windows and water supply pipes to coatings for food cans, epoxy resins, thermal receipt paper, and dental sealants [1,2,3,4]. Due to the ubiquitous nature of this chemical, human exposure to BPA is widespread [5,6].

A growing body of evidence indicates that exposure to BPA can lead to adverse health outcome [7,8]. A review of the health effects of low-level exposures to BPA in animals has been conducted [9], and several recent studies have found evidence of altered fetal prostate and mammary gland development, inhibition of postnatal testosterone production, and changes in neurodevelopment [10,11]. Elevated prenatal exposures to BPA as established from maternal urinary samples have been found to be associated with multiple adverse effects on the infant as well as reproductive outcomes such as preterm delivery and shortened gestational length [12,13,14]. Animal and human studies have also shown that BPA can lead to DNA damage [15,16].

While several large biomonitoring studies have clearly established the near universal exposure to BPA in North America and elsewhere, similar biomonitoring efforts have not been conducted in the Caribbean until 2008. As part of a Canadian Global Health Research Initiative’s (GHRI) Teasdale-Corti grant funded research initiative, a biomonitoring study was conducted to determine prenatal exposures to a number of toxicants ranging from persistent organic pollutants (POPs) (PCBs, organochlorine compounds and polybrominated flame retardant compounds) to commonly used classes of pesticides such as organophosphates, carbamates, phenoxy herbicides, and pyrethroids, two heavy metals mercury and lead, zoonotic infections, and BPA. The findings of these studies have been published elsewhere [17,18,19,20,21,22]. In this paper, we report on the finding of BPA in pregnant women who live in the 10 English-speaking Caribbean countries where this study was conducted.

## 2. Materials and Methods

### 2.1. Study Protocol

**Figure 1:**
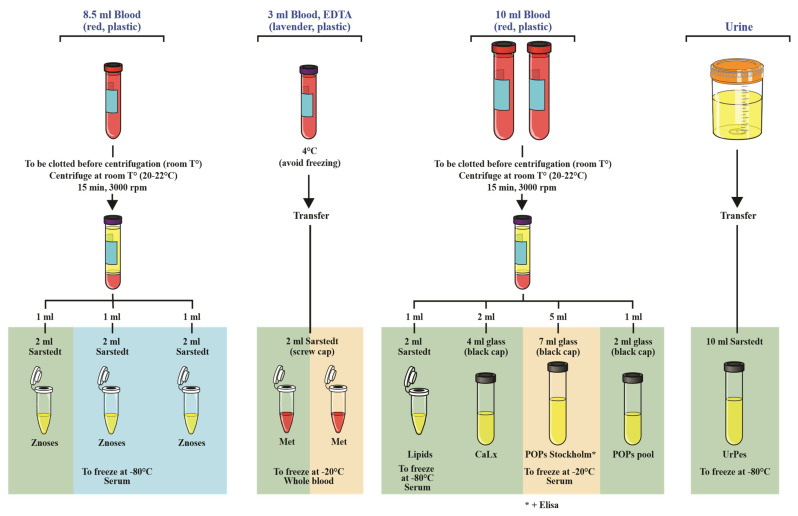

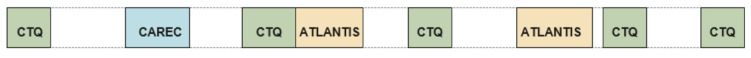
Laboratory analysis schema showing sample processing protocols used to analyze blood and urine samples collected in the Teasdale-Corti grant funded Human Biomonitoring research study. Key: CTQ—Laboratory of Centre de Toxicologie; CAREC—Caribbean Research and Epidemiology Centre, now Caribbean Public Health Agency (CARPHA); ATLANTIS—mobile laboratory.

In addition to ethics approvals secured by the principal investigators of this study from their respective institutions, institutional review board or ethics committee in each of the participating Caribbean countries and governmental approval through the Ministry of Health was first sought and obtained within each Caribbean country. Once all approvals were secured, locally identified nurses and laboratory technicians were trained to recruit pregnant women, obtain their informed consent, and collect biological samples. Collected urine samples were initially poured into 10 mL vials and stored at −80 °C prior to shipping in International Air Transport Association (IATA) certified boxes packed with dry ice to the Centre de Toxicologie (CTQ) laboratory of the Institut National de Santé Publique du Québec (INSPQ) located in Quebec City, Canada, for processing and analysis (Figure 1).

### 2.2. Study Population and Sampling

**Figure 2:**
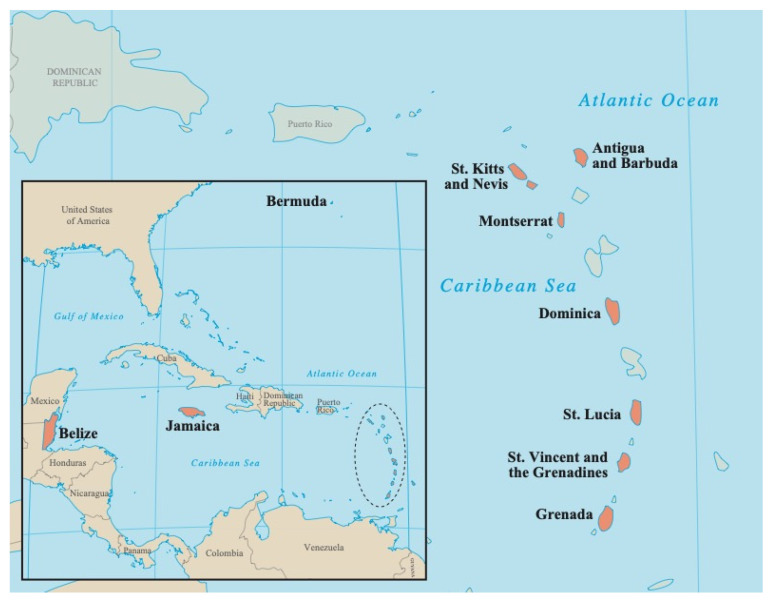
Ten Caribbean countries where BPA samples were collected.

The recruitment and sampling protocols used in this study were modeled on a similar biomonitoring exposure assessment program, the Arctic Monitoring and Assessment Programme (AMAP, http://www.amap.no, accessed on 7 April 2022), which was carried out in circumpolar countries [23]. Following this protocol, pregnant and delivering women ≥18 years coming to the main hospital or health clinics during their last prenatal visits or to deliver were invited to participate in this study by local nurses in 10 Caribbean countries (Figure 2). In most cases, urine samples were taken before delivery; however, in some cases where this was not possible, the sampling was done within two weeks of delivery. In accordance with the AMAP protocol, a sample size of 50 mothers ≥18 years for each country was set. With the exception of Montserrat, 15 samples were randomly selected out of all the samples collected in each island for the presence of BPA. Given the small size of the population in Montserrat, all 15 samples that were collected from this island were analyzed for BPA.

### 2.3. Chemical Analyses

The concentration of BPA was determined by the Centre de Toxicologie du Québec (CTQ) of the Institut National de Santé Publique du Québec (INSPQ) from urine samples. From the samples taken in each island, 15 samples were randomly selected to determine the presence of BPA.

The first analytical step used to measure BPA was the urinary metabolites were hydrolyzed with β-Glucuronidase enzyme. The samples were then derivatized with pentafluorobenzyl bromide at 70 °C for 2 h. The derivatized products were extracted with a mixture of dichloromethane:hexane (8:92). The extracts were evaporated and reconstituted with dichloromethane:hexane (20:80) before being analyzed by GC-MS/MS (Agilent 6890 Network gas chromatograph (GC) (Agilent Technologies; Mississauga, ON, Canada) coupled to a Waters Quattro Micro GC mass spectrometer in tandem (MS/MS) (Waters; Milford, MA, USA) operating in Multiple Reaction Monitoring (MRM) following a negative ion chemical ionization (NCI). The analytical column used was an Agilent HP-5MS 30 m × 0.25 mm i.d. × 0.25 µm film thickness (Agilent Technologies, Mississauga, ON, Canada). The analytical method is described in more detail in [24].

Concentration was reported in units of micrograms per liter (µg/L), and the limit of detection reported (LOD) for BPA of 0.2 µg/L LOD was determined by first estimating the concentrations of analyte yielding a signal to noise ratio of 3. A synthetic urine sample spiked with BPA in concentration ranging from 4 to 10 times the estimated LOD was analyzed (10 replicates) and standard deviation was multiplied by three to obtain the LOD. The intraday precision (repeatability) of the method was 6.6% and the interday precision (reproducibility) was 4.8%.

The internal reference material used to control the quality of the analyses was the in-house reference material from pooled urine samples of exposed people prepared by the Centre de Toxicologie du Québec (CTQ), Institut National de Santé Publique du Québec (INSPQ). The overall quality and accuracy of the analytical method was monitored by the participation in the interlaboratory program as the German External Quality Assessment Scheme (G-EQUAS; Erlangen, Germany). 

### 2.4. Statistical Analyses

The results obtained from each of the sampled Caribbean countries were compared with each other and with comparable data from Canada and the USA. The U.S. data were extracted from the Centers for Disease Control and Prevention’s (CDC) Fourth National Report on Human Exposure to Environmental Chemicals, February 2015, which utilizes biomonitoring data taken from representative U.S. individuals who are part of the National Health and Nutrition Examination Survey (NHANES)(https://www.cdc.gov/exposurereport, accessed on 7 April 2022). From the 2011–2012 NHANES survey, the urinary BPA geometric mean and selected percentiles (in μg/L) of 1230 women were taken and compared with the Caribbean results obtained in this study. In the case of Canada, data from the 2007–2009 Canadian Health Measures Survey (CHMS) for females in the 20–39 years age range were obtained [5]. Additionally, urinary bisphenol A concentrations from ten cities across Canada of women in their first trimester (<14 weeks gestation) reported in the Maternal-Infant Research on Environmental Chemicals (MIREC) study were obtained and used for comparison with the Caribbean results [25]. It is important to note that both the CHMS and MIREC samples were analyzed at the Centre de Toxicologie du Québec (CTQ), Institut National de Santé Publique du Québec (INSPQ), which was the same laboratory used to conduct the analyses for the Caribbean urine samples.

In order to be comparable with the U.S. and Canadian data, the arithmetic and geometric means, lower and upper 95% confidence intervals (CI), the 95th percentile, and the minimum and maximum values recorded were calculated and reported. Additionally, in order to enhance comparability with the U.S. and Canadian results, only those samples where BPA was detected in ≥ 60% (threshold used in the CHMS) of the cases were reported. A value equal to half the LOD was entered for samples with a result below the detection limit.

## 3. Results

From August 2008 to April 2011, 438 maternal urine samples were collected from pregnant women from the 10 Caribbean countries surveyed in this biomonitoring study (Table 1).

Due to budget reasons, 15 urine samples were randomly selected from the approximate 50 samples collected in each island. BPA was detected in 100% of all the Caribbean samples ranging from a low geometric mean of 1.46 μg/L (95%CI 0.86–2.48) in St. Lucia to a high of 4.88 μg/L (95%CI 2.73–8.72) in St. Kitts & Nevis (Table 2).

All sampled Caribbean islands geometric mean concentration levels were higher than those recorded in the two Canadian data sets (1.26 μg/L and 0.80 μg/L) and the U.S. NHANES date set (1.39 μg/L). The 95% Confidence Intervals (CI) for the two Canadian datasets and the U.S. NHAHES (2011–2012) did not overlap with those calculated for Bermuda and St. Kitts & Nevis indicating that the distributions of BPA concentrations in these two Caribbean countries significantly differ from those recorded in North America.

## 4. Discussion

In this paper, we report on the results of a biomonitoring survey of urinary BPA concentration to which prenatal infants in English-speaking Caribbean islands are exposed. Concern regarding the potential effects of BPA exposure to fetuses has been highlighted by several animal studies, which have found a range of negative health impacts ranging from increased susceptibility to mammary cancer to negative impacts on both male and female reproduction and behavior [26,27,28]. Other studies have established that this environmental contaminant crosses the placental barrier and interferes with hormonal, neurological, and immune system development, as well as other physiological functions [29,30].

There are limitations to the interpretations that can be extracted from this biomonitoring study due to the inherent limitations associated with this study’s sampling methodology. Given that a nonrandomized population-based sampling strategy was used, there are potential limitations on the comparability of these results with those obtained from the NHANES, CHMS, and MIREC population-based datasets. Samples were taken only from pregnant and delivering women coming to the island’s main hospital or health clinics during their last prenatal visits or to deliver. However, since the date of conception and eventual delivery are more or less random events, and there is no evidence to suggest that these events are somehow link to BPA exposure, it is likely that the Caribbean BPA urinary samples are defensibly representative of the population from which they were drawn.

Due to budget constraints, with the exception of Montserrat, only 15 of the approximate 50 samples collected in each island were analyzed for the presence of BPA. It should be noted that in most of the Caribbean countries where samples were taken, the population is <100,000 and the majority of deliveries takes place in one or two health care facilities—typically the sole main hospital or sole polyclinic center. In Canada, the proportion of the population sampled in 2010 (34 million) was 1.9 per 100,000 and 5.7 per 100,000 for the CHMS and MIREC studies, respectively. For the U.S., the proportion of the population sampled in 2010 (309 million) was 0.4 per 100,000. With the exceptions of Belize and Jamaica, the proportion of the population sampled in the Caribbean countries was many times higher than that sampled in Canada and the U.S. (Table 2).

Detailed bioinformatic data on the participants were unfortunately not consistently captured in this study. The absence of this data thus does not allow us to explore the characteristics of the women who provided samples to test the homogeneity of the random sampling done in each island and to be able to adequately compare the results to the North American datasets. For example, women who were younger and smoked had significantly higher recorded BPA concentrations, whereas for women in the CHAMACOS study, their maternal age and smoking status were not significant predictors [25,31]. In the MIREC study, the timing of when a woman provided a urine sample was found to be a significant factor with higher BPA concentrations levels recorded for women who provided a urine sample later in the day. Thus, it would be difficult to constructively speculate as to what could be the reason(s) for why St. Kitts & Nevis had a measured BPA geometric concentration level that was about double that recorded in the other Caribbean countries. It should be noted, however, that six of the 15 samples from St. Kitts & Nevis had BPA concentration levels >5 μg/L with two of these recorded BPA concentration values >20 μg/L.

The 95% Confidence Intervals for at least two Caribbean countries––Bermuda and St. Kitts & Nevis––were statistically different from the three North American BPA concentrations datasets they were compared with. Given that overall, the recorded BPA concentration levels from the 10 surveyed Caribbean countries were much higher than those recorded in North America, this is a strong indication that exposure to BPA in the Caribbean remains high. The Canadian MIREC BPA data were captured after the Canadian CHMS 2009–2011 survey readings and were found to be lower (0.80 μg/L vs. 1.26 μg/L) and in a smaller proportion of the sample (88% vs. 97.4%) [25]. This might be a reflection of the laws and regulations that have been passed in these North American countries to ban or strongly discourage their populations from using products that might have BPA in them. In Canada, in 2010 their health regulatory agency issued a ban on the manufacture, sale, or importation of any baby bottles that contain BPA. Furthermore, Canada has listed BPA as a prohibited cosmetic ingredient [25].

While this survey provides an initial baseline measure of maternal BPA concentrations for women living in the Caribbean, the observed variability likely indicates that each island most likely has its own unique exposure profile. This variability of exposure profiles may be due to several factors such as differences in the exposure potential to various sources of BPA as well as differences in each island’s laws and regulations as it relates to the importation and use of products that contain BPA. Hence, a generalizing of the findings from these 10 English-speaking Caribbean countries to all of the other Caribbean countries, which include Spanish-, French-, Dutch-, and other English-speaking countries, should not be done in lieu of individually determining the exposure profiles for each Caribbean.

The validity of comparing the findings from these 10 Caribbean countries with the Canadian survey results is strengthen by the fact that all analyses were done by the same laboratory using the same analytical methodology. Furthermore, the same LODs were used in all of the datasets referenced in this paper.

This study’s BPA biomonitoring data provides baseline data for future studies that over time monitor and evaluate changes in exposure to BPA in this region of the world. Given that BPA containing products are still widely used and that the cumulative neurological and physiological developmental effects of chronic exposures on any fetus will be borne out over the child’s entire lifetime, there is a strong imperative for public health authorities in the Caribbean to encourage their populations—in particular, pregnant women—to raise their awareness of potential sources of exposure to BPA and affect ways to minimize or even eliminate direct contact with this chemical. Healthcare providers should also be educated on the implications that BPA exposures can have on neonates and be provided with materials which they can pass along to mothers on how to curtail such exposures.

There also needs to be legislative efforts taken to enact laws banning the importation and use of products that contain BPA from entering the Caribbean region. To ensure the efficacy of these laws, future biomonitoring surveys will need to be conducted so as to ensure that BPA levels are indeed decreasing and hopefully no longer detected in anyone living in this region of the world.

## 5. Conclusions

This first exploratory biomonitoring study on BPA concentrations in maternal urine samples taken from 10 English-speaking Caribbean countries indicates that prenatal exposure to this reproductive and neurodevelopmental environmental toxicant is taking place throughout the Caribbean region. Given the 100% detection rate and that all of the Caribbean geometric means recorded were higher than those recorded for Canadian and U.S. women, this clearly points to the need for Caribbean governments and public health officials to first engage in legislative and regulatory efforts to ban or minimize the importation and use of BPA products used the Caribbean and, second, to continue to conduct biomonitoring surveys so as to ensure that these laws and regulations are indeed leading to a decrease of BPA concentrations in Caribbean populations.

## Figures and Tables

**Table 1:** Sample size and population characteristics for the 10 Caribbean countries.

Country	Total Population ^1^	Total No. of Samples Collected	Average Age (yrs)	Age Range (yrs)
Antigua & Barbuda	98,728	39	N/A^2^	N/A^2^
Belize	404,915	50	24.4	18 to 36
Bermuda	63,867	50	24.9	18 to 38
Dominica	72,172	47	28.8	19 to 44
Grenada	113,015	50	26.5	18 to 44
Jamaica	2,973,462	47	26.1	18 to 42
Montserrat	5414	15	28.8	19 to 31
St. Kitts & Nevis	53,546	44	N/A^2^	N/A^2^
St. Lucia	184,401	46	29.4	19 to 38
St. Vincent & Grenadines	111,269	50	26.7	18 to 42
Total/Average		438	27	

^1^ Source: The World Bank Open Data (https://data.worldbank.org/indicator/SP.POP.TOTL, accessed 7 April 2022). ^2^ Age of participants was not reported by this country’s data collection team.

**Table 2:** Comparison of 10 Caribbean countries’ urinary bisphenol A (μg/L) in pregnant women with comparable U.S. and Canadian results.

Country	N	Proportion of Population Sampled per 100,000	Arithmetic Mean 95%CI	Geometric Mean 95%CI	95th Percentile	Min	Max	Detection Frequency %
Antigua & Barbuda	15	15.1	2.46 1.38–3.55	1.94 1.34–2.85	7.50	0.60	7.50	100
Belize	15	3.7	3.17 0.67–5.67	1.93 1.11–3.34	19.0	0.20	19.0	100
Bermuda	15	23.5	3.69 1.36–6.01	2.55 1.64–3.97	17.0	1.20	17.0	100
Dominica	15	20.7	4.02 1.82–6.22	2.46 1.34–4.53	14.0	0.30	14.0	100
Grenada	15	13.3	4.51 2.07–6.94	2.46 1.24–4.92	12.0	0.30	12.0	100
Jamaica	15	0.5	2.63 1.26–4.01	1.81 1.11–2.95	8.80	0.50	8.80	100
Montserrat	15	277.0	3.90 1.33–6.47	2.26 1.25–4.07	17.0	0.40	17.0	100
St. Kitts & Nevis	15	28.0	8.34 2.65–14.02	4.88 2.73–8.72	40.0	0.93	40.0	100
St. Lucia	15	8.1	2.38 0.53–4.24	1.46 0.86–2.48	14.0	0.30	14.0	100
St. Vincent & Grenadines	15	13.5	5.14 −2.36–12.65	1.58 0.75–3.34	54.0	0.10	54.0	100
Canada CHMS (Women 20–39)	652	1.9	2.62 2.08–3.16	1.26 1.06–1.49	8.08	-	-	90
Canada MIREC	1936	5.7	-	0.80 0.76–0.85	5.40	-	140.0	88
U.S. NHANES (2011–2012)	1230	0.4	-	1.39 1.25–1.55	8.50	-	-	-

## Data Availability

The data presented in this study are available on request from the corresponding author. The data are not publicly available due to privacy restrictions.
